# Drivers, alternatives, knowledge, and perceptions towards antimicrobial use among Tennessee beef cattle producers: a qualitative study

**DOI:** 10.1186/s12917-018-1731-6

**Published:** 2019-01-07

**Authors:** John E. Ekakoro, Marc Caldwell, Elizabeth B. Strand, Chika C. Okafor

**Affiliations:** 1 Department of Biomedical and Diagnostic Sciences, College of Veterinary Medicine, University of Tennessee, 2407 River Drive, Knoxville, TN 37996 USA; 2 Department of Large Animal Clinical Sciences, College of Veterinary Medicine, University of Tennessee, 2407 River Drive, Knoxville, TN 37996 USA

**Keywords:** Qualitative study, Focus group discussions, Antimicrobial use, Antimicrobial resistance, Veterinary feed directive, Tennessee-beef cattle producers

## Abstract

**Background:**

In recent years, there has been an increased awareness of antimicrobial resistance in both animals and humans, which has triggered concerns over non-judicious antimicrobial use. In the United States, antimicrobial use in food-producing animals for growth promotion or improved feed efficiency is perceived as non-judicious. To facilitate judicious antimicrobial use, the United States Food and Drug Administration implemented the Veterinary Feed Directive, effective from January 1, 2017. Interventions, such as the VFD, designed to ensure the judicious use of antimicrobials among cattle producers may be more effective if the factors that inform and influence producer AMU practices are addressed. The specific objectives of this study were to determine the following among Tennessee beef cattle producers: (1) the most common drivers for using antimicrobials, (2) the perceived alternatives to antimicrobials, (3) the knowledge and perceptions regarding antimicrobial resistance, and (4) the preferred avenues for receiving information on prudent antimicrobial use. A total of 5 focus group meetings with beef producers were conducted in East, Middle, and West Tennessee. Each focus group was video recorded and thematic analysis was performed using NVivo.

**Results:**

The factors that producers considered to drive antimicrobial use were the type of cattle operation, disease and animal welfare, economic factors, veterinarian consultation, producer’s experience and peer support, Veterinary Feed Directive, and perceived drug efficacy. Vaccination, proper nutrition, and other good management practices were considered alternatives to antimicrobial use. To encourage vaccine use among small producers, participants suggested packaging vaccines into smaller quantities. Antimicrobial resistance was perceived to be a problem affecting animal and public health. Participants suggested additional education for cattle producers on the prudent use of antimicrobials as a measure for improving antimicrobial use. The veterinarian, producer associations and meetings, and county extension agents emerged as trusted avenues for channeling information on prudent antimicrobial use to cattle producers.

**Conclusions:**

Several factors drive antimicrobial use among cattle producers in Tennessee. Participants generally perceived their antimicrobial use to be discreet and only when necessary. More awareness of drivers for the development of antimicrobial resistance and continuing education on prudent antimicrobial use is needed for Tennessee beef producers.

**Electronic supplementary material:**

The online version of this article (10.1186/s12917-018-1731-6) contains supplementary material, which is available to authorized users.

## Background

In recent years, there has been an increased awareness of antimicrobial resistance (AMR) in both human and veterinary medicine. This increased awareness has triggered concerns over non-judicious antimicrobial use (AMU) in animals, especially due to the perceived risk associated with the zoonotic transfer of resistant pathogens from animals to humans [1]. Although there is currently no robust evidence concerning the impact of AMU in food animals on AMR in human pathogens, some studies suggest evidence of AMR transmission from food animals to humans, while other studies do not support such transmission [2–4]. This lack of strong evidence has led to an on-going controversial debate on the public health impacts of AMU in food animals [2, 5].

Recent studies have shown that indiscriminate use of antimicrobials for both therapeutic and non-therapeutic purposes in animals leads to propagation and shedding of substantial amounts of AMR microorganisms [6, 7]. Furthermore, antimicrobial treatment failure in swine herds was found to be associated with the use of multiple antimicrobial drugs [8]. Despite the controversies around the public health impacts of AMU in animals, it is necessary that judicious practices are widely adopted by all sectors within the animal agriculture food production system in order to prolong the efficacy of current antimicrobial agents [9].

The World Health Organization (WHO) recommended complete restriction of AMU for growth promotion and disease prevention in food-producing animals to preserve the efficacy of medically important antimicrobials [10]. Judicious approaches to AMU in animals have been supported and instituted in many countries based on the precautionary principle [6, 11]. The precautionary principle is a guiding tenet of public health which recommends adoption of preventive measures in the face of uncertainty and exploring various alternatives to potential threats to public health [12].

In the U.S., AMU in food-producing animals for growth promotion or improved feed efficiency is perceived as non-judicious and use for disease management has minimal veterinary oversight due to lack of food animal veterinarians in some areas [13]. To facilitate the judicious use of medically important antimicrobials in food producing animals, the FDA implemented the Veterinary Feed Directive (VFD), effective from January 1, 2017, authorizing the use of medically important antimicrobials in feed and water for therapeutic purposes under the supervision of a licensed veterinarian. Interventions, such as the VFD, designed to ensure the judicious use of antimicrobials among cattle producers may be more effective if the factors that inform and influence producer AMU practices are addressed. Producers consistently base their decisions and actions on a complex system of core values and knowledge. A review by Garfoth suggested that producers do what makes sense to them in the circumstances of their farms, families, and businesses [14]. Behavioral change communication can be effective in educating the farming public about the dangers of non-judicious AMU if the producers’ knowledge, attitudes, skills, and aspirations about AMU and AMR are considered [15].

Studies conducted on United Kingdom pig farmers and pig veterinary surgeons identified economic factors, issues surrounding farming systems, management, agricultural factors, and external pressures as key drivers affecting AMU [1, 16]. Among New Zealand dairy producers, veterinary advice and the producer’s personal on-farm experience were identified as primary drivers of AMU [15]. However, prior to this study, the drivers of AMU by U.S. cattle producers were not documented. A 2007 quantitative survey of Tennessee (TN) beef cattle producers found that higher AMU was associated with herd size > 50, participation in beef quality assurance or master beef producer certification programs, quarantining of newly purchased animals, use of written instructions to treat disease, and observation of withdrawal times [17]. Nevertheless, this 2007 survey did not use qualitative methods to identify drivers of AMU among beef producers.

The purpose of this study was to identify and document the factors driving AMU, alternatives, knowledge, and perceptions towards AMU among Tennessee beef cattle producers. The specific objectives of this study were to determine the following: (1) the most common drivers for using antimicrobials, (2) the perceived alternatives to antimicrobials, (3) the knowledge and perceptions regarding AMR, and (4) the appropriate avenues for receiving information on prudent AMU. These findings will optimize the efforts of targeted campaigns to apply nationwide stewardship of AMU. These efforts could, in the long run, lead to responsible AMU and the reduction in selection pressures from non-judicious use that drive AMR.

## Results

### Focus group participant characteristics

A total of 39 beef producers, 1 female and 38 male, from a wide range of beef cattle production systems in Tennessee participated in the 5 focus groups. Participants’ perceived ages ranged from late twenties to early seventies. The reported herd size per producer ranged from approximately 20 to 225 cattle (Table 1).

**Table 1:** Focus group participant characteristics

Focus group	Geographic region (location)	Number of participants (*n*)	Herd size range	Gender of participants	Cattle operation type (number of participants)
1	Johnson City, East Tennessee	9	40–80	All male	Cow-calf operation (*n* = 2)
					Cow-calf and backgrounding (*n* = 2)
					Stocker (*n* = 2)
					Backgrounding and finishing (*n* = 1)
					Cow-calf and stocker operation (*n* = 2)
2	Dickson county, middle Tennessee	9	40–135	All male	Cow-calf producer (*n* = 3)
					Cow-calf producer and commercial stocker (*n* = 1)
					Seed stock producer (*n* = 1)
					Stocker (*n* = 1)
					Brood cow producer (*n* = 1)
					Seed-stock and brood cow producer (*n* = 1)
					Seed-stock and replacement bull, heifers (*n* = 1)
3	McNairy county, west Tennessee	8	30–200	All male	Black angus operation (*n* = 1)
					Angus seed-stock operation (*n* = 2)
					Seed stock operation (*n* = 2)
					Cow-calf operation (*n* = 2)
					Cow-calf operation and angus seed stock (*n* = 1)
4	Jefferson county, East Tennessee	8	20–200	All male	Cow-calf operation (*n* = 6)
					Stocker (*n* = 1)
					Cow-calf and backgrounding operation (*n* = 1)
5	Athens, McMinn county, East Tennessee	5	30–225	Male	Cow-calf (*n* = 2)
					Cow-calf and backgrounding operation (*n* = 1)
					Brood cow and backgrounding operation (*n* = 1)
				Female	Cow-calf and backgrounding (*n* = 1)

The degree of similarity between focus group pairs (Jaccard’s similarity index) ranged from 27 to 33%. This Jaccard’s similarity index showed there was diversity among participants in the different focus groups. Percent agreement (in coding) between each pair of coders was > 75%.

### Objective 1: Drivers of antimicrobial use practices

**Fig. 1:**
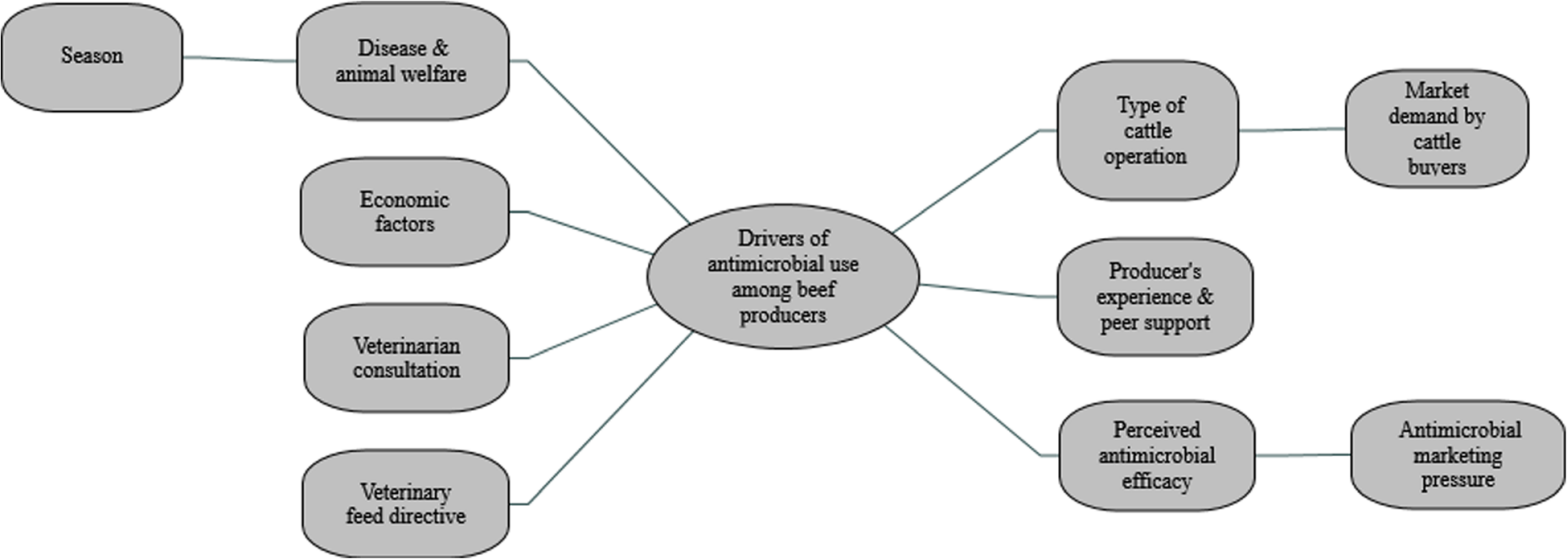
A thematic map showing drivers of antimicrobial use among beef producers in Tennessee, 2017

The major themes identified as drivers of AMU were: a) type of operation; b) disease and animal welfare; c) economic factors; d) veterinarian consultation; e) producer’s experience and peer support; f) VFD; e) perceived drug efficacy (Fig. 1**)**.

A detailed presentation of these factors accompanied by excerpts from the focus group transcripts is given below.

#### 1a. Type of cattle operation (management factors)

The type of operation was associated with the degree of AMU. Stocker cattle operations use more antimicrobials due to stress and potential sickness associated with stocker operations compared to cow-calf operations. Additionally, compared to producers with open herds, those with closed herds require and use less antimicrobials in their operations.

*… a lot of the cattle that we see not just in this county but surrounding counties, 85 to 90% of the cattle are mismanaged cattle. So, if it was left up to the mamma cow, cow-calf operators to take better care and management of their cattle, it would help No. 2 and No. 9’s larger backgrounder or stocker operators, not just on antibiotic cost but health and letting them turn cattle over faster to ship or do whatever… [No. 1, focus group 1].*

*… And with the stocker cattle, used a lot more antibiotics because the cattle required it because of the stress and potential sickness and a lot of the diseases that we go through the cattle … But with the cow-calf operation, unless it’s warranted by some medical condition, they don’t get it … [No. 3, focus group 4].*

#### 1ai: Market demand by cattle buyers

Along with the routine use of antimicrobials associated with a specific type of cattle operation, consumer requests encourage increased AMU. Some buyers request that cattle breeders treat cattle with antimicrobials prior to shipping. This prophylactic treatment is aimed at reducing the risk of infection during transit from the breeder to the buyer.

*… we bring in cattle – I would say quite weekly but almost biweekly from other places. And we sell across the country and ship stuff out. Antibiotics is second nature to us. We have to have that. A lot of people out in California we sell cattle to, they mainly buy young calf and resell it. They want that calf to have draxxin before it gets on the truck, because they don’t know how long it’s gonna take to get them from our ranch in Tennessee to California. They might stop at ten other ranches to water and this and that. And they want a shot of draxxin just for those ten days so that calf don’t pick up anything or get sick on day three and have a seven-day haul to get to where they’re going. I agree with a closed herd, which with my operation, we can’t do that … [No. 8, focus group 3].*

#### 1b. Disease and animal welfare

In order to maintain the welfare of their cattle, producers tend to use antimicrobials for disease management in their herds. The presence of early signs of disease was considered to commence AMU because producers feel they have a responsibility to protect the lives of cattle under their care.

*… We use it as needed sometimes – foot problems. They can step on something, stab or a thorn or something in their foot. And we use antibiotics for that. If a calf in the wintertime acts like he’s getting pneumonia or something like that, we see the early signs – whether it be a cow, calf or whatever, we give that … [No. 7, focus group 2].*

*… If I see early symptoms, I’ll treat early and try to head things off rather than let it get full blown, otherwise, it’s grass and hay, protein and mineral … [No. 3, focus group 2].*

*… As far as what’s important when deciding to use the antibiotics, they key factor comes down to economics and the animal welfare. I think cattlemen are very strong proponents of animal welfare because if the animal is not being treated properly or is not healthy, we’re not making money off of them. And that’s what we have to make sure of at the end of the day … [No. 3, focus group 4].*

#### 1bi: Season

Antimicrobial use for disease management tends be influenced by season (weather/climate). Wintertime use of antimicrobials was mentioned in focus group 1 for the management of interdigital phlegmon and focus group 2 in suspected cases of respiratory disease. However, participants from focus group 4 stated that antimicrobials were mainly required from spring through fall for the management of anaplasmosis and infectious bovine keratoconjunctivitis.

*…*. *Antibiotics depends on the weather. Spring or whether it’s fall …*. *have an issue where you need some antibiotics … [No. 8, focus group 4].*

*… I do use some feed grain antibiotics when I have train wrecks … in September and October. You are going to have some sick cows during what we call dead-cow month October/November… Occasionally, there’s some feed through antibiotics that goes through those wrecks … [No. 2, focus group 4].*

#### 1c. Economic factors

The need to obtain economical gain from a healthy herd was an important driver of AMU among producers. The producers frequently stated that they use antimicrobials to maintain a healthy and productive herd for sustainable economic gain. They were defensive and frequently asserted that antimicrobials are only used when necessary and not indiscriminately, as perceived by policy makers, consumers, and the public.

*… I think it comes down once again to economics … that economic threshold... But as a producer, you have to look at it from an economic standpoint is it worth it to give the antibiotic? Is it worth it to pay the vet bill at this point? Or am I going to try something that’s worked in the past?... [No. 3, focus group 4].*

#### 1d. Veterinarian consultation

Although access to emergency veterinary care was mentioned to be difficult in some areas, a section of participants from areas with active food animal veterinarians (McMinn county, Jefferson county) considered veterinarian consultation an important influencer of AMU. Producers with a good relationship with their veterinarians consulted them on AMU issues.

*…*. *I just work close with my veterinarian. He goes off label or whatever you’re trying to treat at the time. I just stay with that …*. *[No. 6, focus group 5].*

*… I’ll say consultation with a veterinarian is one factor… [No. 3, focus group 4].*

However, for those with limited access to food animal veterinarians, veterinarian consultation was not an influencer of AMU.

*… We don’t have a veterinarian we regularly work with. What [we do] is just visual appraisal if we have sick animals … [No. 7, focus group 4].*

Some producers in East TN, Middle TN, and West TN decried the lack of food animal veterinarians in their areas.

*… [It is] more difficult [to access a food animal veterinarian] than it was a few years ago. Most of them [veterinarians] going to be cat and dog vets. They won’t treat the cattle... [No. 6, focus group 2].*

#### 1e. Producer’s experience and peer support

The participants frequently stated that they rely on their own experience, knowledge, and judgment when deciding to use antimicrobials in their cattle and tapped into the AMU experiences of their peers (other producers). However, in situations that are difficult to handle, they consult the veterinarians. There was a shared belief among participants that peers are easy to access given that some areas do not have food animal veterinarians.

*… I think for most of us, we’re relying on our own experience and our own knowledge. If it’s something that I’ve seen before and I know how to treat it, I’m going to treat it like I treated it before … whatever has been successful. If it happens to be something that I have a question about, I can text one of the vets I was talking about … [No. 4, focus group 2].*

*…*. *experience and not necessarily my experience but experience of producers that’ve done the same thing I’m doing a lot longer than I have. I find a lot of times they know – nothing against the veterinarians, the producers deal with this every day. In a lot of cases, they know more about it than the veterinarian does and will offer some more solid advice of what to use, when to use it, that kind of thing but still consulting with the veterinarian in doing the right thing … [No. 3, focus group 4].*

*… What I pick up on is when I start having wrecks, I just pick up the phone and call somebody else who does the same thing … He’s doing the same thing I’m doing week in and week out … You get on the phone. You start calling. Hey, what’s working? What medicine are you using?... [No. 2, focus group 1].*

#### 1e. Veterinary feed directive

Throughout all the focus groups, it was common for participants to state that the restriction of in-feed antimicrobial products at sub-therapeutic concentrations and for prophylactic indications by way of the VFD has led to increased occurrence of disease in herds and increased mortalities. Examples of those diseases are infectious bovine keratoconjunctivitis, anaplasmosis, and interdigital phlegmon in calves.

*… There is increase in injectable because we’re having a lot more pinkeye, a lot more foot rot. Even in our weaned calves this year, we have foot rot we never had before, never... [No. 3, focus group 5].*

#### 1 g. perceived drug efficacy

Antimicrobials perceived to be more efficacious are often chosen in preference to those perceived to be less efficacious. In the event of treatment failure, producers switch from apparently less effective antimicrobial to a “more effective” one sometimes based on their own observation or on the advice of a veterinarian or their peer group.

*… And the medicines – I don’t know about anybody else, but I’ve used every medicine that’s new and old and come out. And the truth of the matter is one week this might work. The next week, this one don’t work. We have a veterinarian come through all the time that wants you to switch. … Sometimes when you switch, it’s a disaster. I’ve used everything that’s come out … To me it seems like the medicines aren’t strong enough, if anything. They’re not working. We had Draxxin come out a few years ago. I mean, it worked great. Now you just as well shoot farm water at them with a dart gun. That’s what we found out. They just wouldn’t respond to it. … [No. 2, focus group 1].*

#### 1 gi: Marketing pressure from veterinary pharmaceutical companies

Marketing from drug companies tend to shape producers’ perception of antimicrobial efficacy, as well as antimicrobial choice. Producers expressed the marketing techniques to be persuasive and aggressive.

*… I don’t know about anybody else here, but there’s nothing no worse than to look up a driveway and see the Pfizer man coming up the driveway. If they’re like me, they go try to hide because it’s gonna drive you crazy. Their product’s always the best and always this and always that. Most of the time, we wanna get it done. We wanna feed. We wanna make sure the cattle’s healthy... [No. 2, focus group 1].*

### Objective 2: Alternatives to antimicrobials

The commonly mentioned alternatives to antimicrobials used by focus group participants generally included proper animal nutrition, use of good management practices, use of vaccines, and immunostimulants. The excerpts that support these alternatives are provided below.

#### 2a. Proper animal nutrition

Maintaining cattle on good ration, good pasture, and clean fresh water were suggested as prerequisites to a healthy productive animal. Adequate mineral and vitamin supplementation was also considered important in raising healthy animals to abrogate the need antimicrobials.

*… We use good minerals, good feed … [No. 5, focus group 5].*

*… You’ve got to keep your cattle in a good body score. They can’t be too fat, definitely not too skinny. It’s just like No. 7 said, we have good grass, good mineral program and a good vaccination program – not antibiotics, your viral vaccines … [No. 3, focus group 5].*

#### 2b. Good management practices

Good management practices, such as on-farm biosecurity/infection control programs, vector control (tick control), rotational grazing, proper sanitation and hygiene, stress management, provision of good cow comfort through proper housing, and routine deworming of the herd, were suggested as preventive measures to limit AMU. Participants who maintained closed herd operation types stated that a closed herd operation system helped them in preventing disease introduction from other farms and minimized AMU on their farms. However, those with open herds practice isolation of newly introduced animals from other farms to prevent disease introduction and minimize the need for AMU.

*… You do everything management wise to prevent the need for it [need for antimicrobials], whether it be sanitation, nutrition, daily removal of stress from the animal’s life – in your case, trying to keep out infectors from them. We do everything within our power management wise. And it’s a whole program, not just one step … [No.7, focus group 3].*

*… We don’t have a closed herd. Definitely, [we] see the benefits to a closed herd … And we’re doing that in picking new animals along the way. … We isolate a period of time and vaccinate as soon as we get those animals to see if we’ll have any disease and sickness and keep that from being a threat to rest of the herd … [No. 5, focus group 4].*

#### 2c. Vaccination and immunostimulants

Vaccination and use of immunostimulants, such as zelnate®, were frequently mentioned as alternatives to AMU. Also, immunostimulants are used to boost the animals’ immune response to infection.

*… we use vaccines … [No. 5, focus group 5].*

*… we have good grass, good mineral program, and a good vaccination program – not antibiotics, your viral vaccines … [No. 3, focus group 5].*

*… And to go along with vaccinations, the cattle have to be prepared to respond to those vaccines. You can’t give vaccines to sick calves or calves that are not prepared to respond and expect them to respond because it won’t work … [No. 4, focus group 2].*

*… We put ours on a good health protocol. They’re run through … twice a year for vaccines, worming … You’ve got to have a healthy animal for your vaccines to work. If you don’t have a healthy animal to start with, they’re not going to work … [No. 6, focus group 2].*

### Objective 3: Knowledge of AMR and perceptions regarding AMR

Generally, many participants were well informed regarding AMR and perceived it to be a threat to both animal and public health. Participants suggested several measures for containing AMR. A detailed presentation of participants’ knowledge of and perceptions regarding AMR is given below.

#### 3a. Knowledge of AMR

Although many participants had a fair understanding of AMR, it was clear from the discussions that some were uninformed regarding AMR. Some participants associated AMR with prolonged use of the same antimicrobials in the farm. A section of producers believed AMR in cattle pathogens does not exist.

*…Has anybody seen when you give them some antibiotic and they don’t respond? Most of them respond. So, they’re not resistant to it … I think most people here are not convinced that there is animal antibiotic resistance …. I do believe there’s human just because of the abuse of antibiotics… [No. 3, focus group 5].*

#### 3b. Perceptions regarding AMR emergence

A section of participants perceived AMR emergence to be a problem challenging animal and public health. It was voiced that AMR could be occurring in Tennessee cattle pathogens.

*… Unless the medicines are changed, then my opinion the bugs or whatever you want to use as a scientific name, are getting resistant because it’s not doing the same thing. I can’t tell you that [because] I don’t know if they’re weakening the medicine… [No. 2, focus group 1].*

The role played by AMU in livestock on the emergence of AMR was generally disputed by participants. Although some producers thought that other producers could be indiscriminately using antimicrobials and contributing to selection pressure associated with non-prudent use, the focus group participants generally perceived their AMU practices to be prudent. Concerns about over-use in cattle production were generally regarded as unfounded and not evidence-based.

*… Use the same antibiotic for everything – some [cattle producers] do that. They’ve only got one bottle, they’ll just give them a dose it… [Unidentified participant, focus group 5].*

*…As mentioned [we use antimicrobials only as needed], just as needed to treat animals that – whether it’s his foot or respiratory illness or cow or calf needs, something like that but only as needed and usually the least potent thing to do the job … [No. 4, focus group 2.*

Participants frequently mentioned non-judicious use of antimicrobials in human health (and not in livestock) as the key driver of AMR in pathogens affecting humans.

*… There’s been misuse on the human side… [No. 7, focus group 4]. …the humans are taking a lot more than the cattle are taking… [No. 2, focus group 2].*

*…I think they take in what has happened in the human side and try to say that’s happening in the beef side, and it’s not. The human side, ya know, I got a sniffle. I go get a shot. They give me a Z-Pack. And we don’t do the animals like that. They don’t get five rounds of antibiotics a year like some people do … [No. 3, focus group 5].*

#### 3c. Proposed solutions to AMR

The focus group participants suggested a wide range of measures for containing AMR. A brief description of measures suggested by the participants is given below.

#### 3c. I restricted use of medically important antimicrobials

Restriction of the use of medically important antimicrobials in food animals was strongly supported and was perceived to be an important measure for prolonging the efficacy of medically/critically important antimicrobials. Participants suggested that medically important antimicrobials should be reserved for use in humans.

*…I’m pretty concerned about the superbugs you hear about in hospitals and the new bugs that are out there that don’t respond to any antibiotic. I think it’s a pretty big concern for all of us how we’re going to treat some of this in the future. I think there are some common sense approaches we can take, especially some of the types of antibiotics we use that are not necessarily used on the human side. I hope we can identify those and not just restrict all antibiotics because I think there are some that are important to us that aren’t used on the human side… [No. 7, focus group 4].*

*… I think avoiding medically important antibiotics for humans in animal production as much as possible [is important]. [We should] use those antibiotics that are not used for human medicine as much as we can … [No. 3, focus group 4].*

#### 3c. Ii use of sound research

More investment in research on AMR and AMU by federal agencies and development of novel antimicrobial drugs by the pharmaceutical industry was suggested. Additionally, it was suggested that scientific evidence of the link between AMU in livestock and development of AMR in animal and human pathogens should be provided to producers. Such evidence whether pictorial or in video format would trigger behavioral change towards maintenance and adoption of prudent AMU by producers. It was suggested that wide consultations with producers before enacting and implementing policies on AMU in animal production would be useful for wider acceptance of such policies.

*… As far as the results that they get from the research that they do on the certain antibiotic, show the results. They say this does this. This does that. Where’s the proof? Show it to us. Show the farmer what it’s doing. Give us the proof. Let us know what it’s doing. Show us pictures. Show us what to do … [Unidentified participant, focus group 5].*

#### 3c. Iii additional education of producers

Additional education of cattle producers on prudent AMU was frequently suggested by participants to improve AMU in cattle production so that selection pressure from non-judicious use can be reduced. Areas in which additional education for producers is needed include proper management of cattle, farm-level biosecurity to prevent disease, use of antimicrobial cycling/rotation in farms, and encouraging producers to always consult the veterinarians on AMU.

*… I believe education [on AMU] is the key to it all… [No. 6, focus group 3].*

*…Well, I think it would be a good thing to teach us on it [antimicrobial use]. And we’ll use that [the acquired knowledge] for our background and start our program … [No. 8, focus group 4].*

#### 3c. Iv promoting vaccination of animals

The need to promote vaccine use among producers for those diseases that are vaccine-preventable was frequently mentioned as a measure for reducing AMU and minimizing AMR selection pressure. Packaging of vaccines into smaller quantities was suggested to cater for producers with small herd sizes because the currently available livestock vaccines are mainly packaged in large quantities. Such large quantities that may be ultimately wasted are perceived to deter small scale producers from using vaccines.

*… I think we could accomplish a lot with proper vaccination programs in the southeast. In Tennessee, we have a lot of part-time producers that just don’t know or it’s not that important to them to have the proper vaccine protocols. And that’s what leads to the need for all the antibiotics at the doctoring background … [No. 7, focus group 4].*

*… there’s so many producers that …they’re not gonna break into a box that says ten doses to vaccinate three calves. That’s throwing seven doses away. I’m just not gonna do it. I don’t know if we can break this down into smaller doses or something just to get these products [to] more smaller producer [s] … [No. 5, focus group 1].*

#### 3c. V simplified antimicrobial labeling

The current antimicrobial labels and information on the antimicrobial package inserts were perceived to be very technical for producers to comprehend. Thus, participants suggested that antimicrobial drug labels and information in the antimicrobial package insert should be written in non-technical language to make such information easy for producers to comprehend.

*…. Sometimes you read those drug labels. I’m not a chemist or biochemist. But maybe get the veterinary college to simulate the information down to a working level …. [No. 6, focus group 5].*

*... I deal with people every day that try to read those labels and can’t understand them – too many big words. I think if they would speak in plain language, say this is for shipping fever, pneumonia, or what this specifically does. That would be a help for people… [No. 5, focus group 5].*

#### 3c.Vi miscellaneous measures

Other measures suggested for reducing AMU and containing AMR include the promotion of infection control and biosecurity measures; discouragement of veterinary pharmaceutical companies from aggressive marketing of antimicrobial products; training more food animal veterinarians; training para-professionals, such as veterinary technicians; and incentivizing the producers through subsidies so as to encourage wider adoption of use of vaccines and alternatives to antimicrobials.

*… Start at the top with the drug producers…. I would ask them to not be marketing at such an aggressive level as to prevention, cure,* et cetera*,* et cetera *… [No. 3, focus group 2].*

*…. encouraging people to use vaccines. I think the best encouragement is if you hit them in the pocketbook. When everything’s bringing the same price, whether it’s vaccinated or unvaccinated, there’s no motivation for producers to vaccinate. But if there’s some price differentiation, people will spend the $5.00 to vaccinate. We have to make it justified economically, once again … [No. 3, focus group 4].*

### Objective 4: Avenues for receiving information on prudent AMU

Avenues for reaching out to producers on prudent AMU vary by producer’s age as well as the geographical region. Although no one medium for receiving information on prudent AMU would work for all producers, the following were identified as viable avenues: email, farm magazines, feed sales persons, peers/other producers, producer meetings, the veterinarian, county extension agents, photographs, videos, and hard copies mailed to their mail boxes.

*…I love Internet. But I also love hardcopy [as source of information] because [if] I get a magazine, and I won’t read it. I’ll stick it back in the bookcase. Something might come up, and I’ll read it through it and be an article from two years ago. And I can go back and kinda research. I kinda like it both ways… [No. 4, focus group 3].*

However, the veterinarian (for areas with food animal vets), producer associations/meetings, and county extension agents were commonly mentioned as trusted avenues for channeling information on prudent AMU to cattle producers.

*… if there’s information, I want it from a trusted source and not from somebody that I don’t know or somebody just trying to sell something. I trust my vet and other producers who have used products or may know more than I know about it.... [No. 4, focus group 2].*

## Discussion

A deep understanding of factors influencing producers’ decision-making, their beliefs, attitudes, and perceptions is needed as a basis for building effective interventions [14]. Hence, identifying producers’ current behavior towards AMU is a critical step towards achieving success in policy interventions that promote judicious AMU among cattle producers. This qualitative study provides a detailed understanding of drivers of AMU among beef cattle producers in TN. Additionally, this study identified the producers’ alternatives to antimicrobials, their perceptions regarding AMR, and the appropriate avenues for disseminating information on prudent AMU to these producers. These findings should aid in shaping and optimizing interventions that seek to promote and improve judicious AMU in TN and the entire US. The impact of such interventions on AMU could then be validated when measuring AMU both qualitatively and quantitatively.

Our study shows that the factors driving AMU among beef producers in TN are numerous and in conformity with those identified in other studies elsewhere [1, 18]. Occurrence of disease at farm level, cost-benefit analysis of the treatment of disease, producer’s expertise and experience, and producers attitude towards risk, among other factors, have previously been identified as drivers of AMU [18]. Previous European studies have demonstrated that economic factors drive farmers’ AMU [1]. Among dairy cattle producers in New Zealand and dairy producers in South Carolina, owner’s experience was an important driver of AMU [15, 19]. The OIE prudent use guidelines discourages the veterinary pharmaceutical industry from directly advertising antimicrobials to food-animal producers [20]. In the present study, producers perceived the veterinary antimicrobial marketing techniques to be persuasive and aggressive. Aggressive marketing of antimicrobials is a known driver of AMU that has led to calls for banning pharmaceutical industry and drug retailers from advertising antimicrobials [21]. Several findings of our study are in keeping with findings of these previous studies.

The VFD was identified as a key factor that is driving increased use of injectable antimicrobial agents by producers and decreased use of in-feed antimicrobials, since it became effective on January 1, 2017. This is an important finding that needs to be further validated. It is necessary to conduct a targeted country-wide evaluation of the impact of the VFD on the use of injectable antimicrobials in the US. In Denmark, where the use of antimicrobials for growth promotion (AGP) has been banned, the reported impacts of the ban are conflicting. In one study, the ban reportedly led to a reduced total AMU and increased therapeutic use of antimicrobials due to significant increase in health problems in Danish pigs [22]. However, in another study [23] that evaluated changes in AMU and productivity in the Danish pig industry, long term swine productivity was not affected by the ban on AGP use.

Optimal housing and hygiene practices, climate control, feed, and water quality are known to be prerequisites for reduction of AMU in farm animals [24]. In the present study, there was strong appreciation of good management practices and vaccination as alternative approaches to reduce AMU. The WHO action plan to combat AMR has identified vaccination as an alternative to AMU and part of the solution to AMR [25]. The producers’ suggestion for promotion of vaccinations as an alternative to antimicrobials is in line with the WHO action plan to combat AMR. Use of vaccines eliminates the need for antimicrobial therapy and indirectly combats AMR, reducing AMU through indirect protection provided by herd immunity [26]. Countries, such as Denmark, have already taken steps to promote the use of vaccines and to discourage use of antimicrobials, especially critically important antimicrobials (CIAs). Denmark, since 2013, is applying differentiated taxes (0% on vaccines, 0.8% on narrow-spectrum penicillins and other veterinary medicines, 5.5% on other veterinary antimicrobials, and 10.8% on CIAs) on antimicrobials to promote the use of vaccines by farmers [18, 27]. The participants in this study suggested that vaccines should be packaged in smaller quantities to encourage small producers to use vaccines, and incentives should be provided to farmers to encourage the adoption of alternatives to antimicrobials. Further evaluation of the potential benefits of these suggestions would be useful in providing a better justification for their adoption.

A previous study suggested that farmers should be provided with clear evidence of the consequences of non-judicious use of veterinary antimicrobials and the need to reduce AMU [28]. Dissemination of existing knowledge to producers about best practices to reduce AMU while at the same time not compromising animal health and production has been suggested to convince producers of the feasibility of production with less AMU [28]. In the present study, the participants suggested that producers should be provided with scientific evidence that shows how the use of AMU in food animals contributes to AMR. Although many participants had a fair understanding of AMR, others appeared not to be conversant with AMR, with some participants stating that such resistance in cattle pathogens did not exist. These findings suggest a need for more awareness among producers of what constitutes and drives the development of AMR. If producers don’t believe there is AMR in veterinary pathogens, then they are likely to maintain those practices that would select for resistance.

The WHO has suggested restriction of critically important antimicrobials for use in food animals [29]. In this study, some participants were positive about restriction of medically important antimicrobials for use only in humans and suggested that such restriction will be significant in preserving the efficacy of medically important antimicrobials. With more awareness, cattle producers are likely to embrace such AMU restrictions as recommended by WHO.

The participants in this study called for more sound research and development of new antimicrobials. This suggestion echoes well with calls by various actors for industry to develop novel antimicrobials [25, 30]. The participants suggested antimicrobial drug labelling should be made easy for producers to comprehend and should be written in non-technical language. This is an important suggestion that needs to be considered by pharmaceutical companies. In the day-to-day running of farms, it is the farmers themselves and their farm staff who make ultimate diagnostic and antimicrobial treatment decisions for their animals, sometimes under veterinarians’ guidance [15]. The authors contend that simplified drug labels (with non-technical language) might actually reduce the complexity that would cause inaccurate dosage determination by producers. Accurate dosage determination is important for prudent use.

In a UK study, farmers perceived themselves as prudent antimicrobial users [1]. In our study, participants generally perceived their use of antimicrobials to be prudent (responsible and within sound reason) and concerns about antimicrobial misuse/over-use in cattle production to be unfounded and not evidence-based. Such perceptions could likely hinder behavioral change towards prudent AMU. Behavioral change communication to educate the farming public about the dangers of uncontrolled AMU would likely be a challenge, since most producers perceive their practices to prudent. Researchers in Europe found that when producers do not see the need to change behavior, long-established on-farm practices are difficult to change [31]. Possibly, quantification of on-farm AMU and benchmarking best practices could cause producers to critically reflect on their current AMU practices. Nevertheless, campaign efforts targeting behavioral change on AMU among TN producers should focus on encouraging producers to continue benchmarking AMU practices from peers.

In the present study, the veterinarian (for areas with food animal vets), producer associations/meetings, and county extension agents emerged as trusted avenues for channeling information on prudent AMU to cattle producers. In the Netherlands, administration of veterinary antimicrobials is restricted to veterinarians only and farmers are only permitted to administer antimicrobials to their animals in specified cases without the physical intervention of the veterinarian [32]. However in the U.S., most antimicrobial treatments in farms are administered by non-technical farm personnel (producers and farm employees) [33, 34]. In the present study, veterinarian’s prescription was an important driver of AMU only in areas with active food animal veterinarians and training of more food animal veterinarians was suggested due to the shortage of food animal veterinarians in the U.S. Some producers in East TN (Johnson City focus group) and Middle TN (Dickson County focus group) decried the lack of food animal veterinarians in their areas. This lack of food animal veterinarians in some counties in Tennessee could be a key barrier to judicious use of antimicrobials. Also, training of veterinary nurse practitioners and para-veterinarians was suggested to fill the gap of lacking food animal veterinarians. More access to food animal veterinarians could play a key role in stimulating change towards prudent AMU among producers. Although encouraging behavioral change among producers is necessary intervention for promoting prudent AMU and managing AMR, the lack of food animal veterinarians in some counties make it difficult to implement this intervention. Training of food animal para-professionals and licensed veterinary technicians might be worth exploring (although it might emerge as a contentious issue in the veterinary community).

In human medicine, integration of behavioral change messages into routine health care has been suggested as a measure for improving AMU practices [35]. Because the veterinarians, producer associations/meetings, and county extension agents are the trusted avenues for reaching out to producers, targeted behavioral change messages towards prudent AMU could be integrated into routine farm visits and veterinary/agricultural extension programs. The use of behavioral techniques such as motivational interviewing informed by assessing producers’ readiness for change could be useful [36]. Producer meetings/associations could be used to identify AMU training needs and raise more awareness about AMR and prudent AMU among producers. European researchers suggested that AMU behavioral change among producers can be realized if farmers are offered a sense of ownership of the recommendations for judicious AMU [37]. It would be beneficial to conduct studies exploring objectified, reproducible, and transparent methods for quantifying on-farm AMU in the U.S., since such measures could create awareness and stimulate behavioral change towards prudent AMU.

Like any other focus group study, our findings may have been biased by the presence of dominant participants, such that the results may reflect the opinions of the dominant participants, rather than that of the group. However, such bias was minimized by having a moderator in the research team with a behavioral/social science background, skilled in moderating such meetings. Selection bias resulting from purposive sampling may also inevitably be an issue. However, purposive sampling of participants allowed for inclusion of beef producers with experience in different beef cattle production systems and from different geographical areas to represent a range of beef cattle producers in TN. Cluster analysis of the focus groups (Jaccard’s similarity index, ranging from 27 to 33%) suggested that there was great diversity of opinions among participants in the different focus groups. The issue of AMU in farm animals is emotive given the current debate in the media that is shaping the public/consumer perceptions of AMU in food producing animals. Because producers are aware of concerns about non-judicious AMU in animal production, social desirability bias could also be an issue in this study. The producers might have given socially desirable responses. To assess how the factors identified in this study represent the opinions of all beef producers in the state, a quantitative study built on preliminary findings of this study was conducted and findings presented in a separate paper.

## Conclusions

This study provides insight into the several factors that drive the use of antimicrobials among cattle producers in TN. Participants generally perceived their use of antimicrobials to be discreet. However, what the producers perceive as prudent AMU may not necessarily be prudent use. As a result of this study, campaign efforts targeting behavioral change on AMU among producers should focus on encouraging producers to continue benchmarking AMU practices from peers. Benchmarking best practices could perhaps cause producers to critically reflect on their current AMU practices. To reduce the burden of AMR, more awareness of what constitutes and drives the development of AMR, and additional education on prudent use of antimicrobials is needed for beef producers. Training on prudent AMU is likely to be well received by producers if the information comes from their veterinarians, county extension officers, or trusted fellow producers. The trainings should utilize published evidence of the consequences of non-judicious use of veterinary antimicrobials and the need to improve judicious AMU in livestock. Perhaps such training may cause reflection on current practices and would trigger acceptance of messages that aim at behavioral change towards prudent AMU.

## Materials and methods

### Focus group design, structure, and procedure

We conducted a total of five beef producer focus groups in East TN, Middle TN, and West TN in June 2017. Overall, 39 producers participated in the focus group discussions. These regions were chosen based on the demographic density of the Tennessee beef cattle population [38]. For recruitment, the leadership of the Tennessee Cattlemen’s Association (TCA) invited members (via e-mail) with experience in different cattle production systems and from different geographical areas to represent a range of beef producers in TN. All the four authors attended each focus group. Each focus group comprised of 5–9 producers (participants) recruited from a purposive sampling technique and lasted approximately 90 min. An informed consent form giving an overview of the study was provided to all participants, and a signed consent was obtained before their participation in the focus group discussion. Participants could opt out of the discussion at any time, and a meal was provided to each participant as an incentive.

A semi-structured interview guide which was modified after the first focus group was utilized (see Additional files 1 and 2). The modified interview guide (Additional file 2) consisted of 11 open-ended questions. To maintain anonymity, each participant was assigned an identity number, which was used throughout the discussion. Participants announced these numbers before speaking and were identified by these numbers for any follow-up questions. All the focus group discussions were moderated by one of the researchers (EBS) with a background in the behavioral sciences. As described previously, the moderator’s role and responsibility was to give guidance to the discussion and to allow free discussion to develop, while ensuring that all areas in the topic guide were addressed [39, 40]. Three members of the research team (JEE, MC, and CCO) took hand written notes of any key points, provided clarifications to questions, and asked follow-up questions when necessary. At the end of each focus group meeting and before the next focus group discussion, the research team held a debriefing session to allow for discussion of emerging themes and for comparison between focus groups [35]. Data saturation was reached during the fifth focus group discussion. These video-recorded focus group discussions were held either at local restaurants or at county extension centers. Recorded video from each focus group was transcribed verbatim by a professional transcription service provider for thematic analysis.

### Data analysis

The transcribed discussions were analyzed using data analysis software (NVivo qualitative data analysis Software; QSR International Pty Ltd. Version 11, 2017). A recursive six-phase approach (familiarization with the data, generation of initial codes, search for themes, review of themes, definition and naming of themes, and report production) to thematic analysis was performed as described previously [41]. In a brief description of the recursive approach, each member of the team read all transcripts from the focus groups to be familiarized with the data. To visualize patterns in the data, the primary author (JEE) performed a cluster analysis (in NVivo) by grouping focus groups that shared similar words. Jaccard’s coefficient, a statistic that measures similarity between groups by determining the percent of word similarity between groups, was used to assess the degree of similarity for each pair of focus groups. The primary author (JEE) developed a master project with initial nodes identified through consensus at the debriefing meetings and distributed the same to the other authors for individual coding. During the thematic analysis, each author was at liberty to use either the already prescribed coding frame in the master project (theoretical/deductive approach) or to create new nodes independent of the prescribed coding frame (the inductive approach). Thus, each author either added nodes to the master themes or created new themes. After the individual coding, the primary author (JEE) imported the other team members’ coded data into the master project and checked if the themes from the individual coding were related to the coded extracts and all the data transcripts. To ascertain the degree of agreement in the data coding, inter-rater reliability testing was performed in NVivo using percent agreement (JEE, MC, EBS, and CCO). The entire team met twice to review and harmonize the results of the independent coding. Disagreements at the first review and harmonization meeting related to definition and naming of themes were resolved at the second review and harmonization meeting. These themes were refined to identify sub-themes and to ensure that each theme is meaningful and clear but distinct from other themes [16]. Sub-themes that were linked by a common subject area or which related to an overall topic were grouped together, given a unique theme title, and considered as major themes. A thematic map was constructed to review the relationships between minor themes and major themes. The findings are presented in accordance with the consolidated criteria for reporting qualitative studies (COREQ) [42]. The COREQ checklist is provided in Additional file 3.

## Additional files

Additional file 1: The first focus group interview guide (docx). (DOCX 18 kb)

Additional file 2: The modified focus group interview guide (docx). (DOCX 13 kb)

Additional file 3: Consolidated criteria for reporting qualitative studies (COREQ): 32-item checklist. (DOCX 25 kb)

## Abbreviations

AMR: Antimicrobial resistance
AMU: Antimicrobial use
CIAs: Critically Important Antimicrobials
OIE: World Organization for Animal Health
VFD: Veterinary Feed Directive
WHO: World Health Organization
