# Supplementary material for: Drivers, alternatives, knowledge, and perceptions towards antimicrobial use among Tennessee beef cattle producers: a qualitative study
Source: BMC Vet Res. 2019 Jan 7;15:16. doi: 10.1186/s12917-018-1731-6 (PMC6323766; doi:10.1186/s12917-018-1731-6)
Supplement: Supplementary file 2 — The modified focus group interview guide (docx). (DOCX 13 kb) [file 12917_2018_1731_MOESM2_ESM.docx]

**The modified focus group interview guide**

1. What kind of operation do you run?
2. How do you use antibiotics?
3. How does the veterinary feed directive affect your cattle production?
4. How easy is it to access a food animal veterinarian in your area?
5. Who or what influences your decision to start or discontinue the use of antibiotics? Please share things that are important to you when deciding to use antibiotics.
6. What is your opinion about restricting antibiotics for human use only? How would this affect your production practice? What do you believe about antibiotic resistance?
7. What can producers, consumers, veterinarians, and regulatory authorities do in order to make antibiotic use in cattle better?
8. What would you advice the secretary of health and human services to do about the causes and solutions of human and animal antibiotic resistance?
9. Please share other management practices or products besides antibiotics that you use to prevent or treat disease.
10. In your opinion, what specific type of information would you as cattle producers need and like to be receiving about antibiotic use? What is the best format?
11. What is important to you about this topic?
