# Supplementary material for: Drivers, alternatives, knowledge, and perceptions towards antimicrobial use among Tennessee beef cattle producers: a qualitative study
Source: BMC Vet Res. 2019 Jan 7;15:16. doi: 10.1186/s12917-018-1731-6 (PMC6323766; doi:10.1186/s12917-018-1731-6)
Supplement: Supplementary file 3 — Consolidated criteria for reporting qualitative studies (COREQ): 32-item checklist. (DOCX 25 kb) [file 12917_2018_1731_MOESM3_ESM.docx]

**Consolidated criteria for reporting qualitative studies (COREQ): 32-item checklist**

| **No** | **Item** | **Guide questions/description** |  | **Section where found** |
| --- | --- | --- | --- | --- |
| **Domain 1: Research team and reflexivity** | | | |  |
| **Personal characteristics** | | | |  |
| 1 | Interviewer/facilitator | Which author/s conducted the interview or focus group? | All the authors attended all the focus groups. The third author (EBS) moderated the focus group discussions. | Materials and Methods (Focus group design, structure, and procedure), paragraphs 1 &2. |
| 2 | Credentials | What were the researchers’ credentials? | 1^st^ author (JEE): BVM, MVM, PgD, PhD Candidate  2^nd^ author (MC): BS, DVM, PhD, DACVIM  3^rd^ author (EBS): BA, MSSW, PhD  4^th^ author (CCO): DVM, MS, PhD, DACVPM (Epi) | N/A |
| 3 | Occupation | What was their occupation at the time of study? | JEE: Graduate Research Assistant/PhD Candidate.  MC: Assistant Professor, Large Animal Clinical Sciences.  EBS: Director Veterinary Social work/ Clinical Associate Professor.  CCO: Assistant Professor, Epidemiology and Food safety. | N/A |
| 4 | Gender | Was the researcher male or female | Male: JEE, MC, CCO  Female: EBS | N/A |
| 5 | Experience and training | What experience did the researcher have? | JEE: Underwent qualitative research methods training while at graduate school and has experience in veterinary clinical practice, teaching senior veterinary students at a veterinary school.  CM: Has extensive experience in food animal veterinary practice.  EBS: Has wide experience in moderating group meetings.  CCO: Has wide experience in epidemiology and food safety. | N/A for JEE, MC and CCO.  Moderator’s experience mentioned in the discussion, paragraph 10. |
| **Relationship with participants** | | | |  |
| 6 | Relationship established | Was a relationship established prior to study commencement | No relationship was established prior to study commencement. | N/A |
| 7 | Participant knowledge of the interviewer | What did the participants know about the researcher? e.g. personal goals, reasons for doing the research | Prior to the meetings, the participants knew nothing about the researchers. However, at the beginning of each focus group discussion, participants were informed about the purpose of the study as part of obtaining an informed consent prior to commencing with the discussions. | N/A |
| 8 | Interviewer characteristics | What characteristics were reported about the interviewer/facilitator? E.g. bias, assumptions, reasons and interests in the research topic. | Participants were informed that the moderator was a non-veterinarian with a background in the behavioral sciences (social work). | N/A |
| Domain 2: Study design | | | |  |
| Theoretical framework | | | |  |
| 9 | Methodological orientation and theory | What methodological orientation was stated to underpin the study? e.g. grounded theory, discourse analysis, ethnography, phenomenology, content analysis | Researchers were at liberty to use either inductive or the theoretical/deductive approach to thematic analysis. | Materials and Methods: data analysis section. |
| Participant selection | | | |  |
| 10 | Sampling | How were participants selected? E.g. purposive, convenience, consecutive, snowball | Participants were purposively selected. | Materials and Methods (Focus group design, structure, and procedure), paragraphs 1. |
| 11 | Method of approach | How were participants approached? E.g. face-to-face, telephone interview, mail, email | Participant recruitment e-mail was sent to the leadership of the Tennessee Cattlemen’s Association (TCA) who then shared this email with TCA members and then purposively selected the volunteers for this study. | Materials and Methods (Focus group design, structure, and procedure), paragraph 1. |
| 12 | Sample size | How many participants were in the study? | 39 | Materials and Methods (Focus group design, structure, and procedure), paragraph 1. |
| 13 | Non-participation | How many people refused to participate or dropped out? Reasons? | No participant dropped out of the focus groups. | N/A |
| Setting | | | |  |
| 14 | Setting of data collection | Where was the data collected? Home, clinic, workplace? | Data was collected at county extension centers or at local restaurants were the focus groups were held. | Materials and Methods (Focus group design, structure, and procedure), paragraph 2. |
| 15 | Presence of non-participants | Was any one else present besides the participants and researchers? | No | N/A |
| 16 | Description of sample | What are the important characteristics of the sample? e.g. demographic data, date | Sex: 1 female and 38 male  Perceived age: ranged from late twenties to early seventies. Reported herd size: 20 to 225 cattle | Results section: Focus group participant characteristics and summarized in the table 1 provided. |
| Data collection | | | |  |
| 17 | Interview guide | Were questions, prompts, guides provided by the authors? Was it pilot tested? | Yes, interview guide was provided. There was no specific separate pilot testing done. However, the interview guide was modified based on participant comments after the first focus group. | Materials and Methods (Focus group design, structure, and procedure), paragraph 2.  Additional files section: Additional files 1 and 2. |
| 18 | Repeat interviews | Were repeat interviews carried out? If yes, how many? | No repeat interviews were carried out. | N/A |
| 19 | Audio/video recording | Did the researchers use audio or visual recording to collect the data? | Data was video recorded. | Materials and Methods (Focus group design, structure, and procedure), paragraph 2. |
| 20 | Field notes | Were field notes made during and/or after the interview or focus group? | Yes | Materials and Methods (Focus group design, structure, and procedure), paragraph 2. |
| 21 | Duration | What was the duration of the interviews or focus groups? | The focus groups lasted approximately 90 minutes. | Materials and Methods (Focus group design, structure, and procedure), paragraph 1. |
| 22 | Data saturation | Was data saturation discussed? | Yes. Data saturation was reached at the end of the 5^th^ focus group. | Materials and Methods (Focus group design, structure, and procedure), paragraph 2. |
| 23 | Transcripts returned | Were transcripts returned to participants for comment and/ or correction? | No. Participants could not be identified since data was de-identified at collection for protection of human subjects in research. | N/A |
| Domain 3: Analysis and findings | | | |  |
| Data analysis | | | |  |
|  | Number of data coders | How many data coders coded the data | All the four authors coded the data | Materials and Methods: data analysis section. |
| 25 | Description of the of the coding tree | Did authors provide a description of the coding tree? | The coding is described in the manuscript | Materials and Methods: data analysis section. |
| 26 | Derivation of themes | Were themes identified in advance or derived from the data? | Themes were not identified in advance. Final themes presented in the manuscript were arrived at after two review & harmonization meetings to compare individual data coding. | Materials and Methods: data analysis section. |
| 27 | Software | What software, if applicable, was used to manage the data? | NVivo qualitative data analysis Software; QSR International Pty Ltd. Version 11, 2017 was used. | Materials and Methods: data analysis section. |
| 28 | Participant checking | Did participants provide feedback on the findings? | No. Participants were de-identified, hence could not be traced back. | N/A |
| Reporting | | | |  |
| 29 | Quotations presented | Were participant quotations presented to illustrate the themes/findings? Was each quotation identified e.g. participant number | Yes, quotations were presented verbatim (in participants’ own words) to illustrate the themes/findings. Each quotation was identified by participant number, except for a few un-identified participants. | Results section |
| 30 | Data and findings consistent | Was there consistency between the data presented and the findings? | Yes | N/A |
| 31 | Clarity of major themes | Were major themes clearly presented in the findings? | Yes | Results section and summarized in the thematic map. |
| 32 | Clarity of minor themes | Is there a description of diverse cases or discussion of minor themes? | Yes | Results section and summarized in the thematic map. |
